# The Aix-La-Chapelle Treatment of Syphilis

**Published:** 1900-03-03

**Authors:** 


### THE AIX-LA-CHAPELLE TREATMENT OF SYPHILIS.

At a recent meeting of the North London Medical and Chirurgical Society Mr. Albert Ehrmann, in commenting upon a case of syphilis, gave some interesting information in regard to the treatment of that disease by inunction, as carried out at Aix-la-Chapelle. The skin, he said, was generally considered to be in a fit condition for the absorption of mercury on the third or fourth day after the arrival of the patient. The dose used was at first from two to three grammes daily, and this might be increased to four or five grammes if the process was well borne. They did not generally go beyond the latter dose. The two legs were treated on the first day, the two thighs on the second, the chest and abdomen on the third, the back on the fourth, and both arms on the fifth day. The nipples, the navel, and the hairy parts of the body were avoided, as eczema and acne were easily induced in these parts by the applications. The material used was generally the ordinary grey mercurial ointment. The best time for inunction was thought to be the morning, but not unti the skin had become dry from the bath. The presence of moisture in the skin was unfavourable to the absorption of the ointment. A daily bath, lasting half an hour or so, was taken, and every day several glasses of thermal water were drunk. After the twentieth day of the treatment a steam bath was given on alternate days, followed by an ordinary bath and a cold douche to prevent danger from chill. It was not very clear in what way the sulphurous waters acted, perhaps it was by warding off mercurial enteritis, or possibly by converting some of the mercury into an inactive sulphide.

